# Human dignity education and artmaking: addressing student and teacher wellness together

**DOI:** 10.3389/frhs.2026.1854093

**Published:** 2026-06-29

**Authors:** Moira A. Law

**Affiliations:** Department of Psychology, Mount Allison University, Sackville, NB, Canada

**Keywords:** artmaking, human dignity, socio-emotional learning, student, teacher, wellness

## Abstract

With youth well-being at historic lows and recent marked increases in teacher burnout this perspective paper examines the evidence that suggests by “making room” in classrooms and curriculums for artmaking activities coupled with socioemotional learning there may be improvements in teacher and student wellbeing. This paper reviews the literature on artmaking activities and human dignity education in school settings and proposes a novel framework for an inclusive school-based mental health prevention and intervention program for teachers and students. The framework recognizes that youth with subclinical mental health concerns may benefit from lower-intensity programming compared to students experiencing clinical mental health symptoms. Anticipated impacts for teachers and students include improved communication, a greater sense of connection and belonging, meaning making, self-expression, emotional regulation, and improved overall wellbeing. The paper concludes with implementation strategies for piloting this novel mental health prevention/intervention program in varied school settings with innovative collaborations among therapists, homeroom teachers, and artists. Keywords: socio-emotional learning, artmaking, dignity, teacher, student, wellness

## Introduction

Youth mental well-being appears to be at a historic low point (1). It has been estimated that 50% of mental health problems in youth are identified prior to age 14 years (2) providing an impetus for considering school-based interventions to help youth with clinical and subclinical symptoms in early and middle school grades. It is also well established that teachers' wellbeing is in decline (72) in part due to increased workloads, lack of support, and mounting stress (3). Interestingly, interventions to address teacher burnout have been almost exclusively delivered outside of the classroom (4).

Universal school-based prevention programs are increasingly recognized as a crucial avenue for promoting youth wellness and academic achievement (5, 6). With limited resources and access to mental health services, socioemotional learning (SEL) curriculums have been proliferating to address facets known to support youth wellness such as self-awareness, social awareness, and self-management skills (7, 8). Some work in addressing teacher wellness has also been conducted delivering SEL programs during professional development; however, little research has examined if there are detectable positive impacts on teachers' wellness when they teach SEL programs (9). Recently, an early evaluation of the Human Dignity Curriculum (10) reported that teachers felt they were personally benefiting from teaching that SEL program to their students. They also reported they were experiencing the development of a new shared lexicon with their students (11) which is important as clear and open communication in classrooms increases well-being for both teachers and learners (12, 13).

Engaging in artistic pursuits has also been widely associated with enhanced well-being (14, 15). Art-making activities, sometimes referred to as therapeutic art, emphasize the creative process to reduce stress, relax, and express oneself in a non-clinical setting. Whereas expressive art therapy is a structured form of mental health treatment typically guided by a trained professional to help individuals work through psychological trauma or emotional conflicts as they engage in artmaking. Both forms of art intervention could be delivered in the classroom and have the potential for generating positive mental health impacts for both teachers and learners; however, there is little evidence in the published literature of either intervention being offered as a school-based program for mental health (16). On the contrary, there has been a notable decline in art classes, education, and art related activities in many school systems over the past few decades (17, 73). And despite growing public awareness and media interest in the benefits of flow and other benefits of art making (18), there has been little coordinated effort to implement school-based interventions that could provide students and teachers with regular opportunities to engage in hands-on creative activities.

This current paper is organized in the following manner: first it examines the evidence that participation in artmaking activities is beneficial for both children and adults, followed by an introduction to socioemotional learning that notes human dignity education has demonstrated positive impacts on student-teacher connections, communication, creativity, and generating inclusive classrooms. Finally, evidence supporting the importance of program dosage is presented. A new school-based framework for mental health prevention and intervention programming is then proposed; by “creating space” in curriculums, classrooms, and extra-curricular activities for teachers and students to engage in human dignity education and art-making activities together there may be positive impacts on their overall well-being. The paper ends with specific guidelines for piloting the proposed framework in school settings with innovative collaborations with artists, therapists, and educators.

### Artmaking, expressive arts, and wellness

Youth engagement with arts and cultural activities have been consistently associated with lower levels of mental distress, higher levels of life satisfaction, and better mental health functioning (19). A recent meta-analysis found moderate support for a causal relationship between arts engagement and adolescent mental health and wellbeing (20) and both therapeutic art and expressive arts therapy have been found to positively influence mental health in youth and adults (19, 21, 22).

**Art Making Impacts** The mechanisms in which wellness is impacted during art making have been explored through a variety of lenses (22, 23). One pathway for change has been described as the psychological state known as “flow”, which occurs when a person becomes fully immersed in a meaningful, moderately challenging activity that induces a deep focus and distortion of time. Research has shown that activities that involve one's hands can reliably induce this state (24). The tactile, repetitive nature of many crafts can also promote relaxation by calming the nervous system, while the completion of tangible art products can foster a sense of accomplishment (25). Neurobiological studies also indicate that engaging in creative activities can lower cortisol levels and activate reward pathways in the brain, contributing to improved mood and stress reduction (26). Finally, art making has also been found in clinical settings to provide children and adults with time for reflection, opportunities for co-regulation, shared meaning making, and an increased sense of belonging (27).

**Expressive Arts** Similarly, expressive arts therapy can also improve wellness by reducing symptoms of anxiety, depression (15), and trauma-related stress (74), while enhancing self-awareness, self-compassion, and emotional regulation (21, 28). Expressive art projects focus on the psychological meaning behind the art with the goal of processing emotions through prompts from the therapist. This process differs from art making which simply aims for relaxation and creative output with less direction/input from a guide. Additionally, because expressive arts therapy engages multiple sensory and symbolic channels it does not rely on insight or verbal expression which can make it especially effective for individuals who struggle to verbally articulate complex emotions (29). This is important because it makes this mode of therapy an inclusive and accessible intervention for vulnerable youth including those struggling with mental health challenges, special needs students, and immigrant children (30, 31, 71, 75).

### Human dignity, creativity, connections, and inclusive classrooms

Socioemotional learning curriculums have also been helpful in addressing youth wellness (5). More specifically, dignity affirming SEL curriculums have a pedagogical approach that focuses on student empowerment, creativity, identity formation, and self-worth, by recognizing the inherent, inalienable and universal dignity of every person (32). Socio-emotional learning that focuses on human dignity and/or identity formation may be especially effective for elementary school students who are actively developing early self-concepts, while middle school youth are engaged in early stages of identity formation and self-worth issues (33, 34).

For example, the Human Dignity Curriculum (HDC) teaches students about their unique human capacity for creativity and has students engage in meaningful art-making activities as they explore their own identities, understand others, and reflect on their place in the world (10). Engagement with the ideas presented in the HDC have been shown to foster an emerging, meaningful, and shared language between teachers and students, strengthening communication and connection in classrooms, hallways, and outdoor school spaces (11, 35). This is important, as increased connection between students and teachers has been reported to have beneficial effects for both teachers and learners (13, 36–38). Research conducted by Klassen and colleagues (39) found that when teachers experience high relatedness with students it leads to higher levels of engagement, more positive emotions, and fewer negative emotions. Interestingly, they found teachers' need for connection with their peers, i.e., other teachers, was less pressing than their need for connection with their students, yet little attention in policy or programming within schools has been initiated to implement interventions based on these findings.

More broadly, human dignity education has also been found to foster respectful, peaceful, and inclusive learning environments based on students' increased self-awareness of one's shared humanity with others (40) which is important as diversity is expected to continue to increase in classrooms, schools, and communities (41). Education in human dignity may further support inclusivity by countering divisive and dehumanizing rhetoric found in the broader culture by fostering critical thinking and empathy. Similar to work done by Khanahmadova and Jafarov (42) who examined the impact of infusing humanistic values into art making they found students were able to experience and internalize humanistic values in a meaningful way that not only nurtured self-expression and creativity, but also led to increases in empathy, mutual respect, and social cohesion. This is important as youth have been found to be particularly aware of and disturbed by polarizing geo-political events and other mega-trends, i.e., environmental issues, they have little control over (43, 44) and helps young people develop more inclusive social attitudes and respectful communication (45).

### Socioemotional learning and art

Pairing art with other subjects in school is not a new concept (46). Art integration research i.e., where art skills and concepts are embedded in other areas of learning like history or geography, has found improved academic outcomes for diverse learners in creativity, student engagement, and academic achievement (47, 48), as well as noting increases in emotional regulation, helpfulness, and reduced discrimination among pupils (49). Similarly, pairing socioemotional learning with art is also not entirely novel (50, 76) however very few peer-reviewed published papers have reported on the development, implementation, and/or evaluation of the impacts of such a collaborative approach.

One of the few relevant studies found on SEL-art curriculum pairings was a doctoral dissertation that reported on developing a curriculum for elementary aged students that focuses on enhancing socioemotional learning through artmaking activities (51). This newly developed curriculum was based on the results of a thematic analysis conducted with interview data collected from nine elementary school teachers who reported that young students, i.e., grade one, benefit from short lessons and creative outlets to synthesize socio-emotional learning. Similarly, the program *Creating Compassion* combines art engagement with socio-emotional learning for at-risk early elementary children and has documented increases in empathy and self-regulation in an early case study (52).

Studies examining this blend of SEL-art with teachers are also few, however, a recent paper by Lashley and colleagues (53) examined the impact of an arts-based social-emotional learning community among eight teachers preparing to deliver SEL to their students. During this professional development project, the teachers engaged in collaborative artmaking, reflection and community-building. Positive results were noted with teachers' having an enhanced ability to deliver social-emotional learning instruction. No other study was identified that reported on a SEL-art school-based program for teachers and/or students.

### Program dosage

When considering delivery of human services, one-size-fits-all programs are no longer considered best practice (54, 55). Ensuring an appropriate “dosage” of programming is critical when implementing any intervention (56). The Risk-Needs-Responsivity (RNR) framework is a well-validated paradigm, with youth and adults, that emphasizes the importance of matching the amount of treatment to a client's level of risk/need (77). For instance, clients who are assessed to be low-risk may receive services only once a week compared to high-risk clients who may engage in services three to five times a week. Tailored dosage relating to risk has been validated for youth and adults in a variety of clinical settings including correctional facilities and community-based programming (57, 58). Ensuring services are delivered in proportion to risk ensures efficient allocation of resources, as well as stability, immediate monitoring, and better long-term outcomes for high-risk clients (59). Having low risk clients receive lighter intensity of services safeguards against an over delivery of services that could worsen symptomology and eventually increase their risk as harm can accumulate over time (60–62). Hence, it is important to consider best practices when launching pilot projects or full programs in any human service (63–65).

## Discussion: a new framework for teacher-student wellness

This perspective paper proposes that by having teachers and students engage in human dignity education and art marking activities together it may be beneficial in helping to address the youth mental health crisis and the rising burnout rates of teachers. Art making of various types is related to lower levels of mental distress, higher levels of life satisfaction and meaning-making, and better mental health for adults and youth (19–22, 27). Socioemotional learning curriculums have also been helpful in addressing youth wellness (5) with human dignity education supporting identity formation (33, 34) and positively impacting inter-personal connections in the classroom (11, 35, 39, 40, 42, 45).

The combination of art-making activities and human dignity education may be mutually beneficial by increasing trust, communication, meaning-making, and connection between students and teachers thereby supporting overall wellbeing (13, 36–39). Artmaking may also support the integration of knowledge, skills, and attitudes taught in SEL programs (50, Krawjewski, 2023, 46, 51–53), which themselves have documented mental health benefits (5, 8).

Delivering human dignity education with art may further enhance the therapeutic benefits of artmaking by fostering respectful and inclusive learning environments (36, 40) which may in turn increase students' motivation, comfort-level, and confidence in artmaking activities within safe learning spaces. In addition, dignity-affirming SEL curricula that emphasize student empowerment, creativity, identity formation, self-worth, and self-expression, may promote empathy, mutual respect, and social cohesion, all of which can further support engagement in artmaking activities (13, 37, 38, 42). An integrated SEL-art programming model may be particularly effective in supporting identity formation among elementary and middle school youth (33, 34).

**Figure 1:**
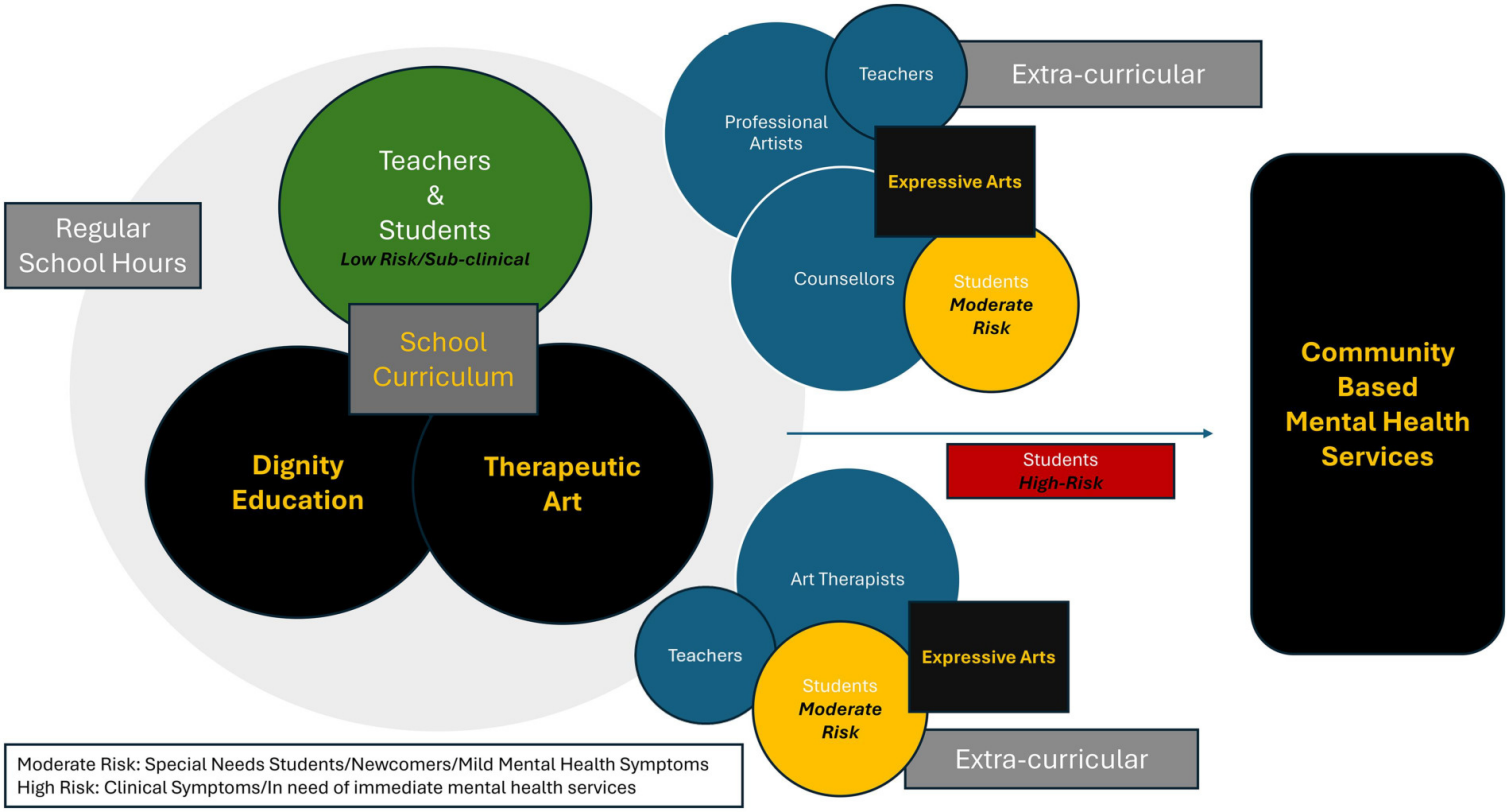
SEL-Art mental health prevention/intervention framework for students and teachers.

When considering the delivery of school-based SEL-art programs for mental health prevention and interventions it is important to distinguish between art-making activities that should be offered to all students, i.e., healthy/subclinical populations, and reserving expressive arts therapy for students with clinical symptomology to ensure appropriate dosage and content is delivered (56, 77). Therapeutic art activities have demonstrated efficacy in promoting wellness by inducing “flow” (24), promoting relaxation (25), lowering cortisol levels and activating reward pathways in the brain (26), and offering opportunities for co-regulation, shared meaning making, and an increased sense of belonging between teachers and students (27). This type of artmaking could be offered to all students at a minimum of once a week in the classroom with homeroom teachers (Figure 1). Conversely, expressive arts activities (which are more therapeutically orientated) should be reserved for students experiencing clinical symptomology, i.e., higher risk students, as it has been found to be effective in reducing symptoms of anxiety, depression (15), trauma-related stress (74), enhancing self-awareness, self-compassion, and emotional regulation (21, 28). This type of artmaking will require professional supports and may be best delivered in extra-curricular spaces for students struggling with mental health challenges, special needs students, and immigrant children experiencing social isolation (30, 31, Li, 2024). These students would need to be screened into the program by a school counsellor/community mental health professional (66) and may require more frequent or sustained engagement with expressive arts therapy, i.e., after school programs. School-based SEL-art education could also prove to be helpful for managing students who are waiting for mental health assessments or on waitlists for services. Hence, by calibrating program frequency, duration, and complexity of the SEL-art program, schools can maximize the therapeutic and developmental benefits of art-making and expressive activities for both clinical and subclinical populations within their student body.

**Piloting SEL-Art Programs** Innovation is hard. Sometimes when new programs are proposed there is resistance, however, there are best practices that can support the introduction and implementation of new initiatives (65). First steps to successful programs depend heavily on strong buy-in from school leadership and teachers. Administrators should be involved early in the planning process, so they understand the goals, resources required, and potential benefits of implementation. Administration will be responsible for ensuring there is support for flexible scheduling, curriculum resources, and any necessary training/supports. Recruiting volunteer teachers to participate in the pilot is equally important, as teachers who choose to be involved are often more motivated, engaged, and provide honest feedback. Allowing teachers to choose their art modality, i.e., painting vs. woodworking, allows them to pursue passion projects with their students. Studies have demonstrated that when teachers engage their passions in the classroom there is increased teacher wellness and retention, while also positively impacting student achievement and wellbeing (67, 68). Creating opportunities for teachers to collaborate, share experiences, and reflect on outcomes throughout the pilot will also increase the likelihood of a successful pilot and eventual school-wide adoption (65).

**School Sites** Larger schools may have more specialized instructors and larger resource networks that could facilitate adoption; however, they may face challenges in creating personalized support/spaces needed for successful implementation. In small schools, closer teacher-student relationships may facilitate adopting a new program more easily, there may be more ease in supervising art-making activities, and students may experience more individualized attention due to lower teacher-learner ratios. However, smaller schools may have fewer resources, smaller budgets, limited course offerings, and less “wiggle room” in scheduling with fewer teachers available for collaboration. In either context, integrating arts-based and dignity-focused interventions has the possibility of positively impacting teacher and student wellness but program details must consider the school's environment to ensure effective delivery of the program (63).

**Collaborations** Only 5% of public-school teachers are art specialists (69). Therefore, expecting art teachers to lead a SEL-Art program is unrealistic, yet many homeroom teachers may feel unprepared to deliver arts-based activities. An interesting study was conducted during the COVID-19 pandemic where researchers had adults engage in simple, low complexity art-making activities such as brushing paints on canvases focusing on brush strokes rather than generating art. Surprisingly, participants were still able to generate flow experiences despite the lack of an individual's skill level or the low level of difficulty of the task. These compelling findings are important and relevant to the proposal in this perspective paper as it offers evidence that flow states can be achieved by teachers and students who don't possess special artistic abilities, knowledge, or skills. As such, the wellness benefits of even primitive art making activities are potentially accessible for the uninitiated and highlights that socioemotional benefits are still accessible with little training (70).

Hence, homeroom teachers should be encouraged to believe that they can deliver effective and enjoyable art making sessions where the goal is flow, relaxation and accomplishment. Partnering with local artists during professional development days, occasional classroom visits, or regularly on a volunteer basis could bring professional creative expertise, confidence, inspiration, and exposure to diverse artistic practices for teachers. Teachers could also support the delivery of extra-curricular expressive arts initiatives under the guidance of art therapists/mental health professionals as they provide (1) continuity in relationship/care by being trustworthy adults already known to students, (2) hold knowledge of students' academic/emotional needs, and (3) could help deliver art activities (Figure 1). School counsellors and/or mental health practitioners in the community may also be able to offer professional oversight and practical insight for responding to the psychological needs of students in such an initiative (66).

**Feedback** Lastly, initiating a pilot project is important as it allows administrators to assess the feasibility of a full-scale implementation. By securing feedback from students, teachers, and perhaps parents, administrators can decide on a manageable number of classrooms to be involved, optimal art making activities, scheduling, and methods to gather regular feedback from teachers and students during early implementation of the full program (64, 65).

## Conclusion

Integrating human dignity education with artmaking activities offers a promising, developmentally appropriate pathway for schools to nurture self-expression, identity exploration, meaning making, and wellbeing among students and their teachers. There is evidence that artmaking in a variety of mediums may support relaxation, emotional processing, and self-expression in the classroom. When combined with human dignity education inclusive learning spaces for all students with a wide variety of types and degrees of vulnerabilities may be generated. Together, arts-based interventions and dignity-centered socio-emotional learning could not only alleviate stress and support mental health but also equip students and teachers with critical skills and attitudes supporting self-awareness, collaboration, and inclusiveness that could eventually make such programs essential components of educational and psychosocial programming in any school setting.

## Data Availability

The original contributions presented in the study are included in the article/Supplementary Material, further inquiries can be directed to the corresponding author.
